# Physiological stress in safer cycling in older age (SiFAr-stress): effect of a multicomponent exercise intervention—a study protocol for a randomized controlled trial

**DOI:** 10.1186/s13063-021-05481-5

**Published:** 2021-08-21

**Authors:** Sabine Britting, Robert Kob, Cornel Christian Sieber, Nicolas Rohleder, Ellen Freiberger, Linda Becker

**Affiliations:** 1 Institute for Biomedicine of Aging, Friedrich-Alexander-Universität Erlangen-Nürnberg, Kobergerstraße 60, 90408 Nuremberg, Bavaria Germany; 2 Department of Medicine, Kantonsspital Winterthur, Winterthur, Switzerland; 3 Department of Psychology, Chair of Health Psychology, Friedrich-Alexander-Universität Erlangen-Nürnberg, Erlangen, Bavaria Germany

**Keywords:** Community-dwelling, Aged, Saliva and hair cortisol, C-reactive protein, Bicycling, Stress, Alpha amylase, Fear of falling, Randomized controlled trial, Perceived stress

## Abstract

**Background:**

SiFAr-Stress investigates the impact of cycling on stress levels in older adults. Uncertainty due to change to motorized bicycle or fear of falling can be perceived as stressors for cyclists. Stress activates different physiological signal cascades and stimulates the hypothalamic-pituitary-adrenal (HPA) axis, which leads to the release of the stress hormone cortisol and further effects such as the development of low-grade inflammation. Both can—in the long term—be associated with negative health outcomes. The aim of the study SiFAr-Stress is to analyze inflammatory processes as well as the activity of stress systems before and after a cycling intervention for older adults.

**Methods:**

In this study, community-dwelling older adults aged 65 years and older will be randomly assigned to either a cycling or a control intervention in a parallel-group design. Objective HPA axis–related measures (saliva cortisol and hair cortisol) will be assessed before, after, and 6–9 months after the cycling and control intervention (T0, T1, and T2). Furthermore, changes in cortisol reactivity in response to the cycling intervention will be investigated at the second and seventh training lessons. Furthermore, secondary outcomes (fear of falling, perceived stress, salivary alpha amylase, and C-reactive protein) will be assessed at T0, T1, and T2.

**Discussion:**

The study will be the first, in which stress- and health-related bio-physiological outcomes will be assessed in the context of a multicomponent exercise intervention, addressing cycling in older adults. It will enable us to better understand the underlying patho-physiological and psychological mechanisms and will help to improve interventions for this target group.

**Trial registration:**

ClinicalTrials.govNCT04362514. Prospectively registered on 27 April 2020

## Administrative information

Note: the numbers in curly brackets in this protocol refer to SPIRIT checklist item numbers. The order of the items has been modified to group similar items (see http://www.equator-network.org/reporting-guidelines/spirit-2013-statement-defining-standard-protocol-items-for-clinical-trials/).

**Table:** 

Title (1)	Physiological Stress in Safer Cycling in Older Age (SiFAr – Stress) – Effect of a multi-component Exercise Intervention: a study protocol for a randomized controlled trial
Trial registration {2a and 2b}.	This study was registered prospectively on 27^th^ April 2020 at clinicaltrials.gov: NCT04362514, https://clinicaltrials.gov/ct2/show/NCT04362514
Protocol version {3}	Version 2.0 dated on July 8^th^, 2021
Funding {4}	internal funding
Author details {5a}	Sabine Britting, Robert Kob, Cornel Sieber, Nicolas Rohleder, Ellen Freiberger, Linda Becker
Name and contact information for the trial sponsor {5b}	The FAU is the sponsor of this trial (investigator-initiated trial). SB and LB are the principal investigators of SiFAr-Stress. SiFAr-Stress is a substudy of SiFAr, of which EF and RK are the principal investigators (contact information: Friedrich-Alexander-Universität Erlangen-Nürnberg, Institut für Biomedizin des Alterns, Kobergerstraße 60, 90408 Nürnberg; sabine.britting@fau.de; Lehrstuhl für Gesundheits-psychologie, Nägelsbachstr. 49a, 91052 Erlangen; linda.becker@fau.de)
Role of sponsor {5c}	SiFAr-Stress is an investigator-initiated trial. Therefore, the sponsor is responsible for planning, conducting, analysis and exploitation of the study.

## Introduction

### Background and rationale {6a}

The demographic change leads to a higher proportion of older persons in the population. Therefore, healthy aging and maintenance of independence are of crucial importance in older adults [1]. Healthy aging is defined as “the process of developing and maintaining functional ability that enables well-being in older age” by the World Health Organization [2]. Positive health and health-related quality of life are highly associated with mobility in older adults [3]. Receiving the ability to live independently and participating in social life requires maintenance of mobility [4–6].

In recent years, cycling as part of daily life mobility has become increasingly popular especially in community-dwelling older adults. Health benefits of cycling [7] as moderate physical activity have been shown in all age groups [8]. Since pedelecs assist cyclists, even more people benefit from this sustainable and environment-friendly locomotion and its positive effect on health and its impact on well-being [7, 8]. In older adults and in middle-aged persons, several studies have shown the beneficial effects of cycling on fitness, cardiovascular health, physical performance, and metabolic processes. Furthermore, cycling in older age can assist in maintaining mobility and, therefore, contribute to living independently and participating in social activities. In contrast, increased bicycle use as well as inappropriate behavior of cyclists and significant traffic growth can result in an increased number of accidents of older adults [1]. Feeling not safe due to road traffic, cycling-related concerns in general, change to motorized bicycle, or lack of practice can be perceived as stressors for cyclists. Until now, no research has been conducted if concerns of cycling can result in chronic stress. Furthermore, the construct of fear of falling (FoF) has been little investigated in the context of cycling-related concerns in older adults so far. Cycling-related concerns address negative outcomes like falls and accidents, and therefore, the construct of FoF (investigated in the context of fall prevention) might be an appropriate way to investigate concerns of cycling.

On the one hand, FoF is associated with several negative outcomes including falls, depression, decreased social contact [9], and reduced quality of life (QoL) [10]. On the other hand, Allali et al. [11] described FoF as a protective mechanism for persons with gait difficulties to avoid activities their motor limitation makes unfeasible. This could mean that cycling concerns result in protective behavior reducing cycling accidents.

Cycling concerns might be associated with chronic stress. Stress arises if individuals are uncertain and lack control about which strategy they should use to cope with the situation [12]. Research on bio-psychological effects of stress found that chronic stress, as well as long-term exposure to anxiety and depressive symptoms, alters the basal activity of the major stress systems [13, 14].

Stress activates different physiological signal cascades, which—in the long term—can be associated with negative health outcomes. Two pathways dominate the acute stress response. The first, which starts immediately after the onset of the stressor, is the activation of the sympathetic nervous system (SNS). This leads to the release of the catecholamines adrenaline and noradrenaline. The second stress response, which peaks with a short delay of a few minutes (about 20 min for socially evaluative stressors) after the onset of the stressor, is the activation of the hypothalamic-pituitary-adrenal (HPA) axis. This leads to the release of glucocorticoids (i.e., cortisol in humans or corticosterone in rodents) from the adrenal cortex. For physical stressors of medium or low intensity (i.e., running, cycling, or strength training) [15–17], only SNS (but not the HPA axis) responses have been reported. However, situations that are associated with anxiety can lead to both, SNS and HPA axis responses. Another physiological stress response that is activated with a delay of a few hours after the onset of acute stressors is the release of pro-inflammatory cytokines (e.g., interleukin-6 (IL-6) and C-reactive protein (CRP)).

Chronic stress alters the basal activity of the major stress systems HPA axis and SNS, which translates to downstream changes in the inflammatory system, ultimately contributing to the development of low-grade inflammation [13]. Therefore, these stress responses are not a health risk factor per se but can lead to negative health outcomes in the long term when people are not able to cope with the stressors. Therefore, chronic stress is associated with an increase in peripheral inflammatory activity [18, 19]. This leads to elevated levels of the stress hormone cortisol and of inflammatory markers such as CRP. Note that for cortisol, very intense and prolonged stress can result in the opposite pattern (i.e., a HPA axis hypo- instead of hyper-activity). However, in the context of daily life stressors, hyper-activity could be expected. Low-grade inflammatory processes might be the link between chronic stress and disease, because stress is associated with low-grade inflammation [13], which is the key factor for morbidity, mortality [20], and predictor for many age-related diseases [21]. In older adults, a state of low-grade inflammation was observed being related with increased plasma levels of pro-inflammatory cytokines such as tumor necrosis factor-α, IL-6, and CRP. These cytokines are associated with an increased risk of muscle strength loss. Physical activity might therefore have a positive effect on muscle loss as well as on chronic stress reduction, which could lead to a reduction of the pro-inflammatory level.

Stress axis activity can be assessed by means of non- or minimally invasive assessment techniques. An established marker for SNS activity is salivary α-amylase (sAA), which can be measured in saliva samples. Salivary α-amylase has been shown to be a well-suited non-invasive marker to investigate SNS reactivity in response to acute stressors. Cortisol levels—as a measure for HPA axis activity—can also be assessed from the saliva. Salivary cortisol has been shown to be associated with plasma cortisol levels and is, therefore, an established marker for HPA axis activity measurements [22, 23]. Collecting bedtime sample on two consecutive days is an accepted and robust way to assess diurnal cortisol output by collecting two saliva samples only. This variable can be used as a marker for general HPA axis activity, which is relatively stable over several days up to a few weeks [14]. A biomarker for systemic long-term stress (up to 3 months, depending on the analysis procedure) is hair cortisol concentration (HCC), which reflects accumulative activity of the HPA axis [24]. Recent or long-term stress is associated with increased hair cortisol levels [25]. Hair is growing constantly about 1 cm per month, which allows HCC to be determined retrospectively over the last months [25].

Inflammatory markers such as CRP levels are usually assessed in blood samples. One minimally invasive procedure is the collection of capillary blood samples by means of dried blood spots (DBS) [26–28]. The DBS method has been shown to be well suited for the assessment of CRP levels [26, 27] and is established in bio-psychological research.

In this study, we investigate the change of inflammatory status as well as stress level in response to cycling as part of physical activity, since in general, physical activity has demonstrated a positive influence on the stress level [29]. The multicomponent exercise program addressing cycling in older community-dwelling persons in the SiFAr study (Siebentritt H, Keppner V, Britting S, Kob R, Rappl A, Sieber C, et al. Safer cycling in older age (SiFAr): a protocol of a randomized controlled trial. BMC Geriatr. 2021. submitted) will be used to directly test whether FoF as a surrogate parameter for cycling-related concerns can be related with the stress system activity and with inflammation. We will test whether in the intervention group FoF, psychological symptoms including stress, anxiety, or depressive symptoms can be reduced and whether a reduction of inflammatory response can be reached. Therefore, cycling in older persons will be investigated from a holistic approach in our study. To the best of the authors’ knowledge, no research has been conducted to investigate the abovementioned pathway.

### Objectives {7}

The aim of the study is to investigate the acute and chronic physiological stress-related health benefits of a cycling intervention in older adults. The analysis of the patho-physiological parameters involves two parts: first, the effects of an 8-week cycling exercise training program on general cortisol and CRP levels and, second, the investigation of the acute physiological effects of a cycling training on cortisol and sAA as stress-related health outcomes. We hypothesize that the cycling intervention will have stress-reducing and health benefits in the long term. Furthermore, we hypothesize that the acute cycling training will be a physical stressor, which will be perceived as a stressor associated with anxiety (leading to HPA axis responses) at the beginning of the training. We expect that at the end of the intervention, the training will be a physical stressor only. This reduced acute stress should lead to a general reduction of cortisol levels.

Therefore, as a primary outcome, the following hypotheses will be tested:

1. Cortisol (measured in the saliva at bedtime and in the hair) will decrease significantly after the intervention in comparison with the control group.

Our secondary hypothesis is that the acute effects of a cycling training on salivary cortisol levels (as a marker for HPA axis activity) will be compared between the beginning and the end of the intervention and will be lower after the seventh than after the second lesson.

Furthermore, we will explore the following parameters to understand better the psychological adaptations to the cycling training: changes in fear of falling and perceived stress before and after the intervention. Moreover, the acute effects of the training on salivary alpha amylase, perceived stress, and anxiety will be investigated.

### Trial design {8}

**Fig. 1:**
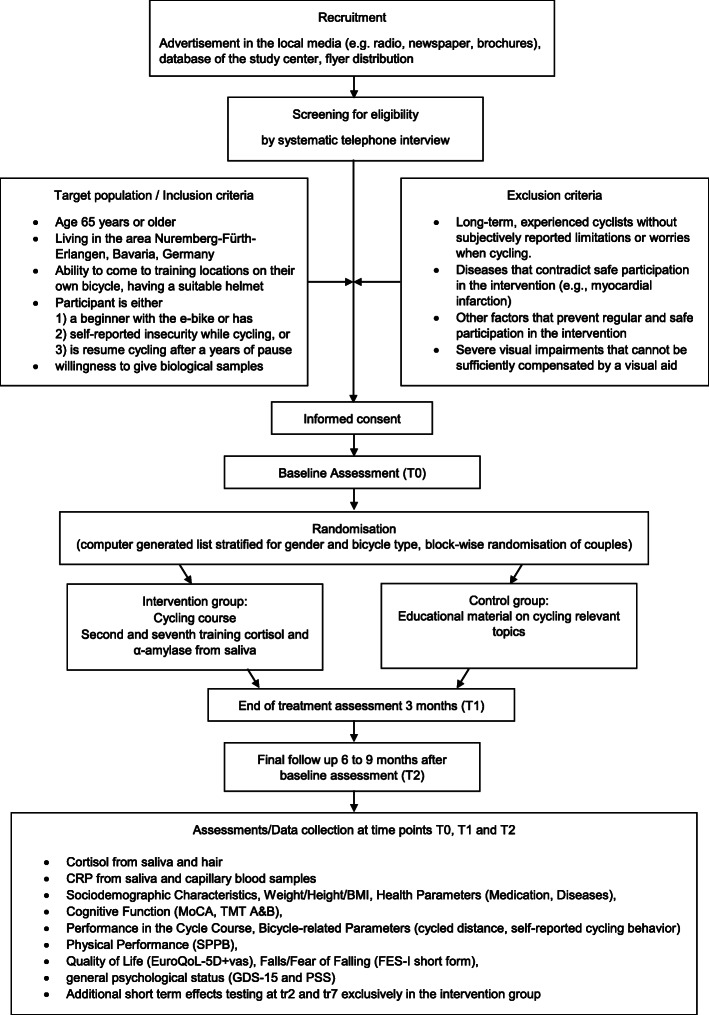
Flow diagram for SiFAr-Stress

SiFAr-Stress is a randomized controlled prospective study with a duration of 3 years and an additional part of the “Safer Cycling in Older Age [SiFAr]” intervention, described elsewhere (Siebentritt H, Keppner V, Britting S, Kob R, Rappl A, Sieber C, et al. Safer cycling in older age (SiFAr): a protocol of a randomized controlled trial. BMC Geriatr. 2021. submitted). SiFAr is addressing the improvement of bicycle-related basic motor competence (e.g., balance, strength, ability to react, cycling skills, and techniques) to enhance cycling competence in older persons. SiFAr-Stress takes the opportunity to investigate and enhance the current knowledge of exercise intervention with regard to the effects on patho-biological level. The flow diagram for SiFAr-Stress is shown in Fig. 1.

## Methods: participants, interventions, and outcomes

### Study setting {9}

SiFAr-Stress is a controlled prospective study with parallel groups. The randomization of participants to an intervention (IG) or a control group (CG) will be stratified. The target populations of this study are community-dwelling older adults who are 65 years and older, who are living in the region of Nuremberg, Bavaria, Germany, and who participate in the SiFAr study. The participants of the main SiFAr study (Siebentritt H, Keppner V, Britting S, Kob R, Rappl A, Sieber C, et al. Safer cycling in older age (SiFAr): a protocol of a randomized controlled trial. BMC Geriatr. 2021. submitted) will be identified by advertising the study in the local media (e.g., radio, newspaper, brochures) and by using a database of the study center. In addition, flyers will be displayed to specifically address potential participants. All participants of the main study will be offered to additionally participate in the sub-study. Participants additionally consent to give capillary blood, hair, and/or saliva samples. The study protocol was approved by the local ethics committee of the Friedrich-Alexander-Universität Erlangen-Nürnberg (number 22_20B). At the Institute of Biomedical Aging (IBA), participants take part in the assessment including questionnaires as well as functional and cognitive tests and capillary blood, hair samples, and saliva samples. All biological specimen analyses will be performed in the laboratory of the Chair of Health Psychology, Friedrich-Alexander-Universität Erlangen-Nürnberg. The study will be conducted in accordance with the Helsinki Declaration.

### Eligibility criteria {10}

The eligibility criteria of the SiFAr-Stress study are shown in Table 1.

**Table 1:** Inclusion and exclusion criteria for SiFAr-Stress

Inclusion criteria	Exclusion criteria
- Age 65 years or older - Living in the area Nuremberg-Fürth-Erlangen, Bavaria, Germany - Ability to come to training locations on their own bicycle, having a suitable helmet - Participant is either: 1) a beginner with the e-bike 2) self-reported insecurity while cycling 3) a re-entrant in cycling - willingness to give biological samples	- Long-term, experienced cyclists without subjectively reported limitations or worries when cycling - Diseases that contradict safe participation in the intervention (e.g., myocardial infarction within the last 6 months, unstable angina pectoris, cerebrovascular event, serious cancer diagnosis, serious neurological diseases like Parkinson, dementia, and other serious new diagnoses) - Other factors that prevent regular and safe participation in the intervention (e.g., prolonged holidays during the training period, alcoholism) - Severe visual impairments that cannot be sufficiently compensated by a visual aid

### Who will take informed consent? {26a}

Participants will give informed consent after reading the participants’ information and after answering all participants’ questions in personal discussion with trained study staff at IBA.

### Additional consent provisions for collection and use of participant data and biological specimens {26b}

For biological specimens, participants sign an additional informed consent, where approval for each sample type, hair, capillary blood, and saliva, as well as for this data retention, is given separately. Conversely, participants are able to exclude one or more biological specimens.

### Interventions

#### Explanation for the choice of comparators {6b}

The control group receives the necessary information about secure cycling without the practical courses. This could be seen as standard care. Several exercise studies analyze the subjective perception and physiological parameters. SiFAr-Stress takes the opportunity to investigate and enhance the current knowledge of exercise interventions with regard to the effects on the patho-biological level.

#### Intervention description {11a}

SiFAr-Stress is an additional part of the SiFAr study following the time course of the main study. SiFAr consists of a practical and a theoretical part within 2 years. In the first year, the IG will participate in the multicomponent exercise program including cycling with a duration of 3 months, whereas the CG will take part in the theoretical course concerning bicycle-relevant lectures. Participants will be recruited from March to July. Both groups (IG + CG) will be tested for the performance in the cycling course and for functional and psychological parameters at T0 (before the intervention), T1 (after the 3-month intervention), and at 6 to 9-month follow-up (T2). To analyze stress- and health-related physiological parameters, biological specimen saliva, hair, and capillary blood will be collected. These patho-biological specimens will be collected during the intervention period to compare the change of different parameters during the intervention. The time points for biological specimen collection are identical as for functional and psychological parameters at T0, T1, and T2. The change of the concentration of stress hormone cortisol in the saliva specimen will be measured at T0 and T1. After completion of the intervention, all participants will be observed and tested as follow-up after 6 to 9 months.

Furthermore, short-term changes of cortisol concentration in the saliva specimens will be monitored for an acute-phase situation (prior, immediately after, and 20 min after the training session). The acute-phase situation will be determined twice during the training period, at the second and the second last training lesson. Therefore, the influence of the exercise training on the acute cortisol response in saliva will be determined.

In addition, hair specimens will be analyzed compared to longitudinal cortisol concentration. The inflammatory marker CRP will be analyzed in the capillary blood. The change in CRP concentration in the blood allows conclusions on inflammatory processes in the body.

### Assessments

The baseline assessment includes general information on sociodemographic variables age, sex, education, living situation, and family status. Anthropomorphic measurements (weight, height), quality of life, health status, tests for physical performance, and functional tests as well as psychological and cognitive status are performed at baseline as well as at follow-up. In addition, objective and self-reported information on cycling habits will be obtained.

#### Assessments of biomedical, anthropometric, and functional health variables

##### Health status

In order to record the health status, the number of diseases and medication will be determined by questionnaire.

##### Recent fall experiences

A fall will be defined as an unexpected event in which the participant comes to rest on the ground, floor, or lower level. We will assess whether participants have experienced a fall event in the 12 months prior to enrollment by a standardized questionnaire used in all our previous studies. If a fall has been taken place, we will ask for the severity of the injuries.

##### Physical performance

Physical performance will be assessed via the Short Physical Performance Battery (SPPB). The SPPB is a brief performance battery based on a set of functional tasks related to the lower extremities. It includes static balance tests, timed short distance walk, and repeated chair stands and hence can be used to assess how well older persons perform simple movements that represent the building blocks of daily activities that require good lower extremity function. Each of the three test domains is scored from 0 (inability) to 4, and a sum score of the three domains is obtained for each participant (0–12). The battery has been used in many studies, and it has a high predictive validity and prognostic value [30]. In addition, one leg stand is performed.

##### Health-related quality of life (HRQoL)

HRQoL will be measured with the EURQOL (EQ-5D) which has been used in the IBA in many studies. The EQ-5D has very good measurement properties and is widely used [31]. The sum score and visual analog scale (VAS, 0–100) will be determined. HRQoL is lately regarded as a critical outcome in contemporary medical research.

### Assessment of cycling related parameters

#### Frequency of bicycle use

A bicycle computer will measure kilometers driven within the 3 months of the intervention period.

#### Questionnaires related to cycling behavior

Cycling behavior will be assessed by questionnaires concerning bicycle type, cycling behavior, mobility biography, and history of accidents.

### Baseline assessment of general psychological status

#### Depressive symptoms

Age-related declines in mobility and functional health tend to be associated with symptoms of depression. We will therefore use the Geriatric Depression Scale (GDS-15), which is appropriate for the age group in our project and was shown to have good sensitivity and specificity [32, 33].

#### Perceived stress

In addition to depression, we will also assess general perceived stress. The perceived stress scale (PSS) is the gold standard for the assessment of sub-chronic life stress in adults [34, 35], and has been validated for older adults as well [36]. We will use the 10-item version as a global assessment of perceived stress, with which the sum scores could be assessed on a scale between 0 and 40. Higher scores indicate higher perceived stress with cutoff points 0–17 reflecting low and 17–40 high perceived stress [34] for older adults aged 60–69 years and 0–15 reflecting low and 15–40 high perceived stress for older adults aged 70 years or older.

### Assessment of fear of falling

#### Fear of falling

As a measure of fear of falling, we will use the current gold standard, the short FES-I scale, which is a 7-item questionnaire related to concerns about falling during basic and more demanding daily activities among independent, community-dwelling older persons. We will use the validated German version of the FES-I [37]. We will analyze the mean change in the sum score (7–28 points) between baseline and 3 months follow-up. Delbaere et al. established cutoff points, 7–8 low concern, 9–13 moderate concern, and 14–28 high concern [38].

### Assessment of cognitive functions

#### Mild cognitive impairment (MCI) screening

The Montreal Cognitive Assessment (MoCA) will be used as a screening tool for MCI. The MoCA yields sum scores between 0 and 30 and is a high-sensitive screening tool [39]. Sum score 26 and higher is considered to be normal, and ≤ 25 indicates cognitive impairment.

#### Executive functions

The Trail Making Test (version A and B) of the CERAD will be used to monitor executive function and mental flexibility since this test was shown to be sensitive to a variety of neurological impairments and processes [40]. In this test, the time and number of faults are monitored.

### Assessment of stress and stress-related pathophysiological processes

#### Stress system activity

Chronic stress has a profound effect on the HPA axis. We will assess the basal activity of the HPA axis using non-invasive, at-home saliva samples measuring salivary cortisol, for example, documented in [41] as well as the collection of hair cortisol samples.

During the first appointment, participants will receive a home sampling package containing detailed instructions, two salivettes (Sarstedt, Nümbrecht), and a pre-stamped envelope for returning samples. In brief, at each time point during the study (T0, T1, and T2), participants were instructed to collect saliva specimens by means of salivettes at two consecutive days prior to bedtime. One hour before saliva sample collection, participants were asked not to eat, drink (except water), smoke, or brush their teeth. Salivettes are kept and moved in the mouth for at least 1 min without chewing on it. During saliva collection, participants will be asked to rate their subjective stress perception. For this, established protocols and instructions that have been used in previous research on older adults will be used [42]. Participants collect the bedtime samples at home and store them in the fridge until all the saliva samples are collected and sent them by postal mail to the study center. Immediately after arrival, saliva samples will be stored at − 30 °C for later analyses. Salivettes will be thawed and centrifuged at 2000*g* at 4 °C. Cortisol concentration in nmol/L will be analyzed in the salivary specimen using commercially available high-sensitive ELISA (Enzyme-linked immunosorbant assay).

Hair sample collection is carried out as previously described by Greff et al. [43]. In brief, hair specimens are cut as close to the scalp as possible after sectioning off the hair at the posterior vertex. A sample of at least 3 cm of length is collected in order to cover a 3-month time period. After extraction, HCC will be measured by ELISA [43, 44]. At the time of hair sample collection, a questionnaire is answered to obtain information on hair-related factors influencing hair steroid hormone concentrations. The questionnaire includes hair washing frequency, application of hair dye, or any other chemical treatment.

On the second and seventh training lessons (tr2 and tr7), participants will provide three saliva samples at three time points (before the training, immediately after the training, and 20 min after the training). Furthermore, perceived stress and anxiety will be rated on 10-point Likert scales, which have been used in previous research at the Chair of Health Psychology [45–48]. From the saliva samples, sAA and cortisol will be assessed as measures for SNS and HPA axis activity. The concentration of sAA will be measured with an enzyme kinetic assay, as previously described [41, 49], and salivary cortisol using a high-sensitive ELISA provided by IBL International according to the manufacturers’ instructions.

#### Systemic inflammation

Systemic low-grade inflammation will be measured by the CRP concentration of capillary blood specimens. Dried blood spot samples will be collected by a minimal invasive finger stick. Therefore, drops of capillary whole blood samples are collected on filter paper and dried [27]. Concentrations of CRP in the blood specimen are carried out by high-sensitive ELISA as described by McDade et al. [26].

A trained study nurse collects all biological specimens as part of the assessment.

### Repeated assessments at T1 and T2

All measures taken at baseline will be repeated at T1 and T2 except for sociodemographic variables. Therefore, changes of variables over a period of 3 months and after a follow-up period of 6 to 9 months will be analyzed.

### Criteria for discontinuing or modifying allocated interventions {11b}

Criteria for discontinuing the intervention for a given participant are adverse event–like accident with severe injuries (e.g., fractures), serious illness, and quarantine (coronavirus) preventing to participate in the intervention.

### Strategies to improve adherence to interventions {11c}

At the first appointments at IBA, the personal meeting includes explanation and practical instruction of saliva sampling procedure as well as a detailed set of written instructions, and a brief questionnaire asking participants for sampling times for reference at home is provided. In addition, participants receive a prepaid envelope to return saliva samples. Close communication via phone calls will be applied, if saliva samples were not returned after a certain period. For the assessments and testing, participants receive personal reminder calls, if desired.

### Relevant concomitant care permitted or prohibited during the trial {11d}

Many factors are known to potentially influence saliva cortisol measurement, like tooth brushing, eating, drinking caffeinated drinks, and ordinary physical activity. We therefore ask participants to avoid eating, drinking (except water), smoking, chewing gum, and brushing teeth for at least 1 h before saliva sampling.

### Provisions for post-trial care {30}

Not applicable

### Outcomes {12}

The primary outcome is the change in cortisol and between the beginning of the intervention (T0) and after the intervention (T1). As secondary outcomes, the mean changes in fear of falling (FES-I) and psychological parameters (sAA, CRP, stress (PSS)) over a period of 3 months (after the intervention) and after a follow-up period of 6 to 9 months will be analyzed. Furthermore, the acute effects on cortisol levels during a session at the beginning and at the end of the training intervention (Tr1 and Tr7) will be assessed.

Cycling accidents during the intervention period present a potential risk for the participants and therefore might be a potential harm outcome.

### Participant timeline {13}

The participant timeline is shown in Table 2.

**Table 2:** Example template of recommended content for the schedule of enrolment, interventions, and assessments*

	Study period				
	Enrolment baseline	Intervention period			Post-allocation
Timepoint**	***T***_**0**_	***t***_***r*2**_	***t***_***r*7**_	***T***_**1**_	***T***_**2**_
**Enrollment**					
**Eligibility screen**	X				
**Informed consent**	X				
**Allocation**	X				
**Study arms**					
***Intervention group***	X	X	X	X	X
***Active control group***	X			X	X
**Assessments**					
***Sociodemographic characteristics***	X				
***Weight/height/BMI***	X			X	X
***Health parameters (medication, diseases)***	X			X	X
***Cognitive function (MoCA, TMT A&B)***	X			X	X
***Performance in the cycle course***	X			X	X
***Physical performance (SPPB)***	X			X	X
***Quality of life (EuroQoL-5D + VAS)***	X			X	X
***Falls/fear of falling (FES-I short form)***	X			X	X
***Bicycle-related Parameters (cycled distance, self-reported cycling behavior)***	X			X	X
***Biological specimen***					
***Saliva samples***	X	3*x*^1^	3*x*^1^	X	X
***CRP (capillary blood sample)***	X			X	X
***Hair sample***	X			X	X

*MoCA* Montreal-Cognitive Assessment, *TMT* Trail Making Test, *SPPB* Short Physical Performance Battery, *VAS* visual analog scale, *FES-I* Falls Efficacy Scale-International Version, *CRP* C-reactive protein

^1^Saliva samples only for intervention group

### Sample size {14}

For statistical analyses of the primary and secondary outcomes, 2-factorial repeated measures ANOVAs are planned (see {20a}). A power analysis for this statistical procedure has been conducted for sample size estimation (GPower 3.1.9.2). Due to the heterogeneity of older persons, the effect size (ES) in this study is estimated to be small to moderate (*f* = .3). With an *α* = .05 and power 1 − *β* = .80, the estimated sample size needed for this effect size is approximately *N* = 111 for a between-group comparison between IG and CG with a 1:1 allocation and three measurement time points. Based on an estimated drop-out rate of 20%, a sample size of *N* = 140 will be adequate for the main objective of this study and should allow for the expected drop-out rate.

### Recruitment {15}

Participants will be recruited exclusively from the SiFAr study’s participants. At IBA, established strategies for recruitment are advertisement in local media (e.g., radio, newspaper, brochures) and using an in-house database of the IBA. In addition, flyers are displayed to bicycle organizations and bicycle dealers to specifically address potential participants. SiFAr will have to recruit almost double of participants in the same time, which allows to meet the number of 140 for SiFAr-Stress according to a participation rate of the last year.

## Assignment of interventions: allocation

### Sequence generation {16a}

A statistician, who was otherwise not involved in the planning of the study design, generated computer-based randomization lists. The criteria for randomization of participants to IG or CG are gender and bicycle type (e-bikes/unmotorized bicycle), whereas couples are block-wise randomized to ensure the participation together in one group.

### Concealment mechanism {16b}

The recruiting team has no access to the randomization list, and the randomizing person receives only the stratification information to assign the intervention.

### Implementation {16c}

After the assignment of the informed consent and the baseline assessments by trained study staff, randomization is performed by enrolling participants in concealed randomization lists by a separate person that only receives the information necessary for stratification.

## Assignment of interventions: blinding

### Who will be blinded {17a}

All biological specimens are labeled by a participant code only, which allows blinding during laboratory analysis.

### Procedure for unblinding if needed {17b}

Not applicable

## Data collection and management

### Plans for assessment and collection of outcomes {18a}

After completion of data collection for each time point, data entry will be performed contemporary to ensure identification of missing data and any discrepancies. This enables to record missing data or wrong inputs from participants. All data will be cleaned, inspected for outliers, and tested for normality of distribution prior to hypothesis testing. Composite scores, e.g., for questionnaires, will be computed where appropriate. Quality control for laboratory data is performed first by using standard procedures and second including intra- and inter-assay variability. Laboratory values not complying with quality control requirements will be repeated. The means and standard deviations for all variables will be computed, and preliminary analyses testing for age and gender effects will be performed.

### Plans to promote participant retention and complete follow-up {18b}

Participants receive reminder calls and invitations to appointments to promote participants completing the intervention and follow-up assessments. Furthermore, each participant receives own data at the end of the study.

### Data management {19}

In general, we will follow the data policy by FAU, released December 2019, regulating data management. All data will be handled adhering to the current German and European data protection laws. Ethics approval has been obtained by the Ethics Committee of Friedrich-Alexander-Universität Erlangen-Nürnberg, Germany, #22_20B, June 22, 2020, and all data collection, storage, and protection procedures were evaluated by the local data protection authority (“Datenschutzbeauftragter der FAU”). All data will be pseudonymized, i.e., a numerical code will be generated and used as a data identifier. Participants will be granted the right to ask for their data to be deleted as long as data is not completely anonymized. One year after the completion of data analyses, all personal information will be destroyed. De-identified data will be stored on secure file servers for 10 years according to DFG recommendations. Data entry will be double-checked by a trained study nurse of the IBA to guarantee correctness, completeness, and plausibility of the provided information.

### Confidentiality {27}

Personal information will be stored in a password-protected data file, separate from all other data.

### Plans for collection, laboratory evaluation, and storage of biological specimens for genetic or molecular analysis in this trial/future use {33}

All biological specimens will be collected at the IBA and stored at room temperature (HCC) or at − 30 °C (saliva and blood samples) as described above. After completion of sample collection for one time point, laboratory analysis will be conducted at the laboratories of the IBA and the Chair of Health Psychology. All samples will be stored until the end of the study for potential re-analyses.

## Statistical methods

### Statistical methods for primary and secondary outcomes {20a}

Data analysis will be performed using descriptive statistical analysis and inferential statistics. The sample data will be presented by frequencies or percentages, means ± standard deviation or median and interquartile range, and graphics. The IBM® SPSS® Statistics for Windows, version 26 software (IBM Corp., Armonk, NY, USA) will be used for all statistical analyses.

For the evaluation of primary outcomes, 3 × 2 analyses of variance for repeated measurements (ANOVA) will be used: For evaluating the long-term changes in CRP and cortisol (hair and saliva), 2-factorial ANOVAs with the factors “time point” (T0, T1, T2) and “group” (IG or CG) will be conducted. For the evaluation of the acute effects of the training intervention, further ANOVAs with the factors “time point” (before, after, and 20 min after the training) and “training number” (second or seventh training) will be calculated. For the evaluation of further secondary outcomes, comparable analyses will be conducted.

### Interim analyses {21b}

Not applicable

### Methods for additional analyses (e.g., subgroup analyses) {20b}

Not applicable

### Methods in analysis to handle protocol non-adherence and any statistical methods to handle missing data {20c}

Non-adherent participants will be excluded from the analysis. Attendance of the participants will be monitored weekly to administer adherence to the intervention. If participants attended at least 6 of the lessons, they are considered as “adherent” to the intervention.

### Plans to give access to the full protocol, participant-level data, and statistical code {31c}

Participant’s data and statistical code will be made available on reasonable request after the publication of the study results.

## Oversight and monitoring

### Composition of the coordinating center and trial steering committee {5d}

Not applicable

### Composition of the data monitoring committee, its role, and reporting structure {21a}

Not applicable

### Adverse event reporting and harms {22}

Adverse events, e.g., cycling accidents, will be recorded.

### Frequency and plans for auditing trial conduct {23}

Not applicable

### Plans for communicating important protocol amendments to relevant parties (e.g., trial participants, ethical committees) {25}

The study protocol was approved by the ethics committee vote (number 22_20B) of the Friedrich-Alexander-Universität Erlangen-Nürnberg and complies with the Declaration of Helsinki and Good Practice Guidelines.

### Dissemination plans {31a}

The results of this study will be disseminated in national and international journals as well as in conference contributions.

## Discussion

### Potential risks and limitations

Due to the COVID-19 pandemic, the intended number of participants might not be reached. To meet this challenge, we established a hygiene concept, which allows performing the assessments. Cycling lessons will take place outside allowing almost contactless saliva sampling for acute phase measurements. SiFAr will have to recruit almost double of participants in the same time, which allows to meet the number of 140 for SiFAr-Stress according to a participation rate of the last year.

The number of hair specimen might be insufficient to reach statistically valid analysis, because short hairstyle, thinning hair, or be bald prevail among community-dwelling older adults. In this case, hair specimen sampling is impossible and only CRP and salivary cortisol will be evaluated for these participants.

Many factors are known to potentially influence saliva cortisol measurement, like tooth brushing, eating, drinking caffeinated drinks, and ordinary physical activity. We therefore ask participants to avoid eating, drinking (except water), smoking, chewing gum, and brushing teeth for at least 1 h before saliva sampling, but they might not always adhere to this request.

For biological samples, informed consent will be obtained individually for saliva, hair, or capillary blood samples or all of these together. Participants will not be excluded from the substudy, if not all three samples can be obtained. Information on medication is collected of all participants, and all participants with glucocorticoid medication will be excluded from the cortisol analysis. Oral intake of glucocorticoids influences the cortisol measurement. To limit this potential risk factor, information on medication is collected from all participants, and all participants with glucocorticoid medication will be excluded from the cortisol analysis. Cortisol levels over 100 nmol/l will be excluded as outliers, which indicates an oral intake of glucocorticoids. These saliva specimens will be exclusively analyzed for alpha amylase.

Intake of medication containing beta-blockers influences the alpha amylase measurement and will be excluded for the alpha amylase analysis and exclusively used for cortisol measurement. If hair specimen sampling is impossible, only CRP and salivary cortisol will be evaluated for these participants.

A large number of factors may influence PSS as well as cortisol concentration: age, sex, smoking, BMI, ever diagnosis of depression, ever diagnosed cardiovascular disease, and time of day.

### Clinical and practical implications

In this study, health- and stress-related bio-physiological outcomes will be assessed in older cyclists, and underlying patho-physiological mechanisms will be better understood. The gained knowledge will help improving interventions for this target group. Since cycling is practiced worldwide, the results will be generalizable to populations from other countries. Furthermore, cycling (among other factors) might contribute to reduce low-grade inflammation (CRP level) and therefore might have a positive influence on health in older age as well as the stress level.

## Conclusions

The study will be the first, in which health- and stress-related bio-physiological outcomes will be assessed in the context of a multicomponent exercise intervention addressing cycling in older adults. It will enable to better understand the underlying patho-physiological mechanisms and will help to improve interventions for this target group.

## Trial status

SiFAr-Stress is in the recruitment stage when this manuscript is completed. Recruitment of participants started in June 2020. This study protocol is version 2.0, dated July 8th 2021. The end of the trial is expected in July 2022.

## Abbreviations

CG: Control group
DBS: Dried blood spots
FES-I: Falls Efficacy Scale International
FoF: Fear of falling
GDS: Geriatric Depression Scale
HCC: Hair cortisol concentration
IG: Intervention group
MCI: Mild cognitive impairment
MoCA: Montreal Cognitive Assessment
PSS: Perceived Stress Scale
SiFAr: Safer Cycling in older Age
VAS: Visual analog scale

## References

[1] Lang JE, Anderson L, LoGerfo J, Sharkey J, Belansky E, Bryant Let al. The prevention research centers healthy aging research network. Prev Chronic Dis 2006;3(1):A17. PMC1500966 16356370

[2] WHO. Global strategy and action plan on ageing and health (2016-2020). Geneva; 2016. https://www.who.int/ageing/WHO-GSAP-2017.pdf.

[3] Davis JC, Bryan S, Best JR, Li LC, Hsu CL, Gomez C, Vertes KA, Liu-Ambrose T (2015). Mobility predicts change in older adults’ health-related quality of life: evidence from a Vancouver falls prevention prospective cohort study. Health Qual Life Outcomes.

[4] Valdes-Badilla PA, Gutierrez-Garcia C, Perez-Gutierrez M, Vargas-Vitoria R, Lopez-Fuenzalida A (2019). Effects of physical activity governmental programs on health status in independent older adults: a systematic review. J Aging Phys Act.

[5] Rejeski WJ, Brawley LR (2006). Functional health: innovations in research on physical activity with older adults. Med Sci Sports Exerc.

[6] Walston J, Hadley EC, Ferrucci L, Guralnik JM, Newman AB, Studenski SA, Ershler WB, Harris T, Fried LP (2006). Research agenda for frailty in older adults: toward a better understanding of physiology and etiology: summary from the American Geriatrics Society/National Institute on Aging research conference on frailty in older adults. J Am Geriatr Soc.

[7] Oja P, Titze S, Bauman A, de Geus B, Krenn P, Reger-Nash B, Kohlberger T (2011). Health benefits of cycling: a systematic review. Scand J Med Sci Sports.

[8] Bauman AE, Rissel C (2009). Cycling and health: an opportunity for positive change?. Med J Aust.

[9] Scheffer AC, Schuurmans MJ, van Dijk N, van der Hooft T, de Rooij SE (2008). Fear of falling: measurement strategy, prevalence, risk factors and consequences among older persons. Age Ageing.

[10] Adamczewska N, Nyman SR (2018). A new approach to fear of falls from connections with the posttraumatic stress disorder literature. Gerontol Geriatr Med.

[11] Allali G, Ayers EI, Holtzer R, Verghese J (2017). The role of postural instability/gait difficulty and fear of falling in predicting falls in non-demented older adults. Arch Gerontol Geriatr.

[12] Peters A, McEwen BS, Friston K (2017). Uncertainty and stress: why it causes diseases and how it is mastered by the brain. Prog Neurobiol.

[13] Rohleder N (2014). Stimulation of systemic low-grade inflammation by psychosocial stress. Psychosom Med.

[14] Miller GE, Chen E, Zhou ES (2007). If it goes up, must it come down? Chronic stress and the hypothalamic-pituitary-adrenocortical axis in humans. Psychol Bull.

[15] Hill EE, Zack E, Battaglini C, Viru M, Viru A, Hackney AC (2008). Exercise and circulating cortisol levels: the intensity threshold effect. J Endocrinol Investig.

[16] Kindermann W, Schnabel A, Schmitt WM, Biro G, Cassens J, Weber F (1982). Catecholamines, growth hormone, cortisol, insulin, and sex hormones in anaerobic and aerobic exercise. Eur J Appl Physiol Occup Physiol.

[17] Becker L, Semmlinger L, Rohleder N. Resistance training as an acute stressor in healthy young men: associations with heart rate variability, alpha-amylase, and cortisol levels. Stress. 2020. p. 1–13. 10.1080/10253890.2020.1799193 32744460

[18] Kiecolt-Glaser JK, Preacher KJ, MacCallum RC, Atkinson C, Malarkey WB, Glaser R (2003). Chronic stress and age-related increases in the proinflammatory cytokine IL-6. Proc Natl Acad Sci U S A.

[19] Rohleder N, Marin TJ, Ma R, Miller GE (2009). Biologic cost of caring for a cancer patient: dysregulation of pro- and anti-inflammatory signaling pathways. J Clin Oncol.

[20] Harris TB, Ferrucci L, Tracy RP, Corti MC, Wacholder S, Ettinger WH, Heimovitz H, Cohen HJ, Wallace R (1999). Associations of elevated interleukin-6 and C-reactive protein levels with mortality in the elderly. Am J Med.

[21] Couzin-Frankel J (2010). Inflammation bares a dark side. Science..

[22] Putignano P, Dubini A, Toja P, Invitti C, Bonfanti S, Redaelli G, Zappulli D, Cavagnini F (2001). Salivary cortisol measurement in normal-weight, obese and anorexic women: comparison with plasma cortisol. Eur J Endocrinol.

[23] Kahn JP, Rubinow DR, Davis CL, Kling M, Post RM (1988). Salivary cortisol: a practical method for evaluation of adrenal function. Biol Psychiatry.

[24] Faresjo A, Theodorsson E, Chatziarzenis M, Sapouna V, Claesson HP, Koppner J (2013). Higher perceived stress but lower cortisol levels found among young Greek adults living in a stressful social environment in comparison with Swedish young adults. PLoS One.

[25] Staufenbiel SM, Penninx BW, Spijker AT, Elzinga BM, van Rossum EF (2013). Hair cortisol, stress exposure, and mental health in humans: a systematic review. Psychoneuroendocrinology..

[26] McDade TW, Burhop J, Dohnal J (2004). High-sensitivity enzyme immunoassay for C-reactive protein in dried blood spots. Clin Chem.

[27] McDade TW (2014). Development and validation of assay protocols for use with dried blood spot samples. Am J Hum Biol.

[28] Danese A, Caspi A, Williams B, Ambler A, Sugden K, Mika J, Werts H, Freeman J, Pariante CM, Moffitt TE, Arseneault L (2011). Biological embedding of stress through inflammation processes in childhood. Mol Psychiatry.

[29] Stults-Kolehmainen MA, Sinha R (2014). The effects of stress on physical activity and exercise. Sports Med.

[30] Freiberger E, de Vreede P, Schoene D, Rydwik E, Mueller V, Frandin K (2012). Performance-based physical function in older community-dwelling persons: a systematic review of instruments. Age Ageing.

[31] Lutomski JE, Krabbe PF, Bleijenberg N, Blom J, Kempen GI, MacNeil-Vroomen J (2017). Measurement properties of the EQ-5D across four major geriatric conditions: findings from TOPICS-MDS. Health Qual Life Outcomes.

[32] Wancata J, Alexandrowicz R, Marquart B, Weiss M, Friedrich F (2006). The criterion validity of the geriatric depression scale: a systematic review. Acta Psychiatr Scand.

[33] Marc LG, Raue PJ, Bruce ML. Screening performance of the 15-item geriatric depression scale in a diverse elderly home care population. Am J Geriatr Psychiatry. 2008;16(11):914–21. 10.1097/JGP.0b013e318186bd67. 10.1097/JGP.0b013e318186bd67 PMC2676444 18978252

[34] Klein EM, Brahler E, Dreier M, Reinecke L, Muller KW, Schmutzer G (2016). The German version of the perceived stress scale - psychometric characteristics in a representative German community sample. BMC Psychiatry.

[35] Cohen S, Kamarck T, Mermelstein R (1983). A global measure of perceived stress. J Health Soc Behav.

[36] Ezzati A, Jiang J, Katz MJ, Sliwinski MJ, Zimmerman ME, Lipton RB (2014). Validation of the perceived stress scale in a community sample of older adults. Int J Geriatr Psychiatry.

[37] Dias N, Kempen GI, Todd CJ, Beyer N, Freiberger E, Piot-Ziegler C (2006). The German version of the falls efficacy scale-international version (FES-I). Z Gerontol Geriatr.

[38] Delbaere K, Close JC, Mikolaizak AS, Sachdev PS, Brodaty H, Lord SR (2010). The falls efficacy scale international (FES-I). A comprehensive longitudinal validation study. Age Ageing.

[39] Nasreddine ZS, Phillips NA, Bedirian V, Charbonneau S, Whitehead V, Collin I (2005). The Montreal cognitive assessment, MoCA: a brief screening tool for mild cognitive impairment. J Am Geriatr Soc.

[40] Tombaugh TN (2004). Trail making test a and B: normative data stratified by age and education. Arch Clin Neuropsychol.

[41] Rohleder N, Nater UM (2009). Determinants of salivary alpha-amylase in humans and methodological considerations. Psychoneuroendocrinology..

[42] Pretscher A, Kauzner S, Rohleder N, Becker L. Associations between social burden, perceived stress, and diurnal cortisol profiles in older adults: implications for cognitive aging. Eur J Aging. 2021. 10.1007/s10433-021-00616-8. 10.1007/s10433-021-00616-8 PMC8563879 34786017

[43] Greff MJE, Levine JM, Abuzgaia AM, Elzagallaai AA, Rieder MJ, van Uum SHM (2019). Hair cortisol analysis: an update on methodological considerations and clinical applications. Clin Biochem.

[44] Xiang L, Sunesara I, Rehm KE, Marshall GD (2016). A modified and cost-effective method for hair cortisol analysis. Biomarkers..

[45] Becker L, Schade U, Rohleder N (2020). Activation of the hypothalamic-pituitary adrenal axis in response to a verbal fluency task and associations with task performance. PLoS One.

[46] Becker L, Schade U, Rohleder N (2019). Evaluation of the socially evaluated cold-pressor group test (SECPT-G) in the general population. PeerJ..

[47] Becker L, Rohleder N (2019). Time course of the physiological stress response to an acute stressor and its associations with the primacy and recency effect of the serial position curve. PLoS One.

[48] Becker L, Rohleder N (2020). Associations between attention and implicit associative learning in healthy adults: the role of cortisol and salivary alpha-amylase responses to an acute stressor. Brain Sci.

[49] Bosch JA, de Geus EJ, Veerman EC, Hoogstraten J, Nieuw Amerongen AV (2003). Innate secretory immunity in response to laboratory stressors that evoke distinct patterns of cardiac autonomic activity. Psychosom Med.

